# Supplementary material for: Evidence supported by Mendelian randomization: impact on inflammatory factors in knee osteoarthritis
Source: Front Med (Lausanne). 2024 May 28;11:1382836. doi: 10.3389/fmed.2024.1382836 (PMC11165061; doi:10.3389/fmed.2024.1382836)
Supplement: Supplementary file 1 [file Data_Sheet_1.ZIP › data/GCST90274830_5e6/830.leaveoneout.pdf]

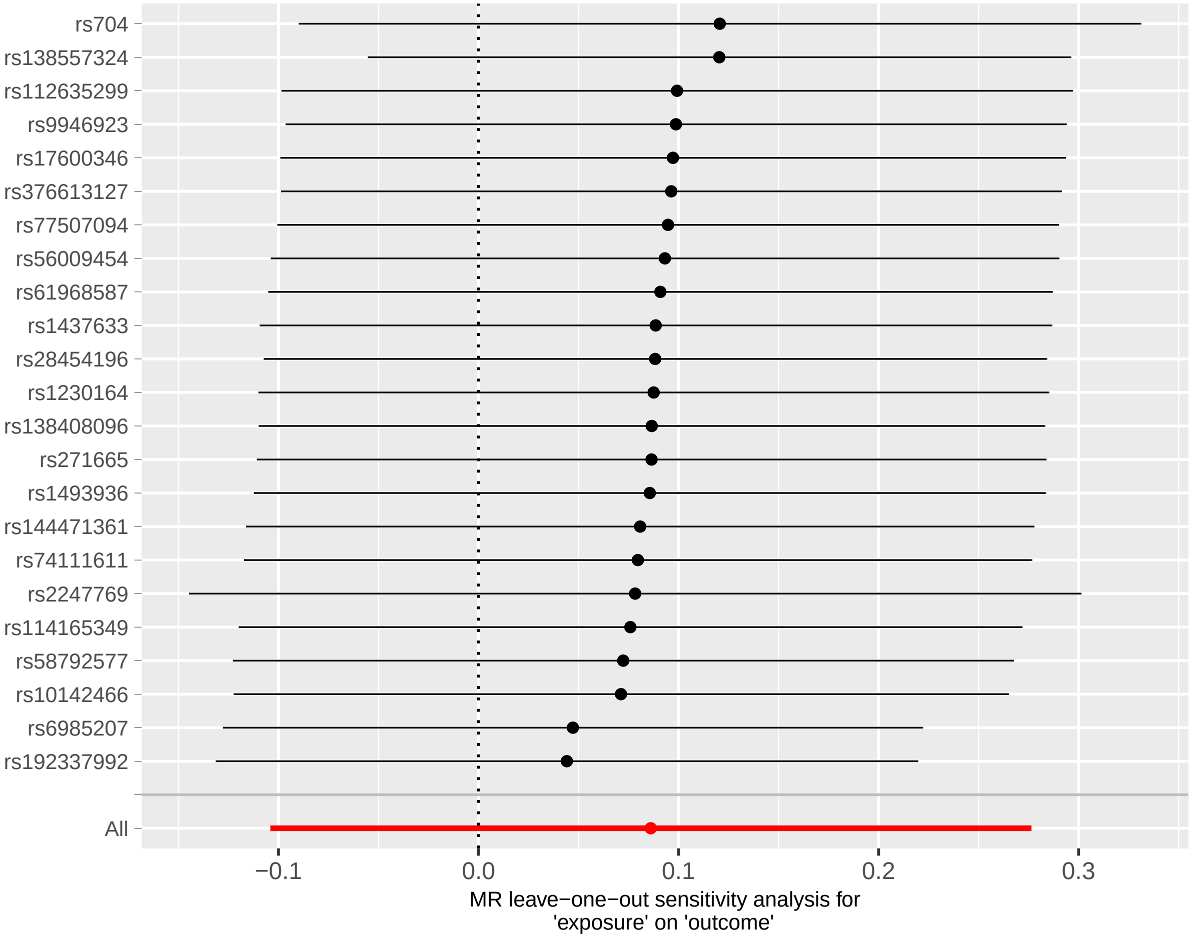

rs704
rs138557324
rs112635299
rs9946923
rs17600346
rs376613127
rs77507094
rs56009454
rs61968587
rs1437633
rs28454196
rs1230164
rs138408096
rs271665
rs1493936
rs144471361
rs74111611
rs2247769
rs114165349
rs58792577
rs10142466
rs6985207
rs192337992
All
−0.1
0.0
0.1
0.2
0.3
MR leave−one−out sensitivity analysis for
'exposure' on 'outcome'
